# Inverting social innovation to transform health system responses to climate change adaptation and mitigation in the global south

**DOI:** 10.3389/fpubh.2024.1333163

**Published:** 2024-05-13

**Authors:** Tarun R. Katapally, Jasmin Bhawra

**Affiliations:** ^1^DEPtH Lab, Faculty of Health Sciences, Western University, London, ON, Canada; ^2^Hirabai Cowasji Jehangir Medical Research Institute (HCJMRI), Pune, India; ^3^Department of Epidemiology and Biostatistics, Schulich School of Medicine and Dentistry, Western University, London, ON, Canada; ^4^Lawson Health Research Institute, London, ON, Canada; ^5^CHANGE Research Lab, School of Occupational and Public Health, Toronto Metropolitan University, Toronto, ON, Canada

**Keywords:** big data, citizen science, climate change, digital health, digital transformations, global health, global south, health systems

## Abstract

Systems thinking is aimed at understanding and solving complex problems that cut across sectors, an approach that requires accurate, timely, and multisectoral data. Citizen-driven big data can advance systems thinking, considering the widespread use of digital devices. Using digital platforms, data from these devices can transform health systems to predict and prevent global health crises and respond rapidly to emerging crises by providing citizens with real-time support. For example, citizens can obtain real-time support to help with public health risks via a digital app, which can predict evolving risks. These big data can be aggregated and visualized on digital dashboards, which can provide decision-makers with advanced data analytics to facilitate jurisdiction-level rapid responses to evolving climate change impacts (e.g., direct public health crisis communication). In the context of climate change, digital platforms can strengthen rapid responses by integrating information across systems (e.g., food, health, and social services) via citizen big data. More importantly, these big data can be used for rapid decision-making,a paradigm-changing approach that can invert social innovation, which we define as co-conceptualizing societal solutions with vulnerable communities to improve economic development with a focus on community wellbeing. However, to foster equitable and inclusive digital partnerships that invert social innovation, it is critical to avoid top-down approaches that sometimes result when researchers in the Global North and South collaborate. Equitable Global South–North partnerships can be built by combining digital citizen science and community-based participatory research to ethically leverage citizen-driven big data for rapid responses across international jurisdictions.

## Introduction

Globally, the frequency and severity of climate change-related weather events, including heat waves, cyclones, and droughts, are exacerbating existing health and social inequities (1). These inequities are particularly apparent among vulnerable and disadvantaged groups in the Global South (1–12), especially in terms of living standards and human health (13–15). Irrespective of the geographic region, the increasing burden on existing infrastructure, lack of resources, and financial constraints continue to prevent vulnerable populations from effectively managing the varied health risks of climate change (16–19). While there is an immense global effort to address climate change, the most negatively impacted communities are often not well represented in these critical conversations. In particular, Indigenous and racialized communities living in the most severely impacted areas are not always included in decision-making regarding mitigation and adaptation efforts (20–25). Moreover, some of the most vulnerable populations are living in densely populated regions of the Global South, with limited resources for climate change adaptation (5, 26–28)—a result of continuing historical injustices driven by centuries of colonization (28, 29).

The impacts of climate change on human health are complex, as they are linked to systems both within and outside of healthcare. For instance, the Intergovernmental Panel on Climate Change (IPCC) reports that the Global South is at a high risk of losing agricultural productivity (30–32) due to climate change (26, 33). Agricultural productivity is linked with various human health outcomes (i.e., the risk of communicable and non-communicable diseases) as well as living conditions (34). Climate change events, in general, have both direct and indirect impacts on health. These impacts include increased risk of heat stroke and cardiovascular stress during heatwaves (35), malnutrition from increasing food insecurity (36), and vector-borne infectious diseases due to frequent flooding (37). Climate change is also influencing mental health outcomes, with issues ranging from ecoanxiety and post-traumatic stress disorder to depression and suicidal ideation (38).

Globally, while health emergencies due to climate change have increased across all populations (27–29, 39), evidence clearly shows a disproportionate impact of climate change on vulnerable populations such as rural communities that rely on agriculture for livelihood (2, 3, 5, 40). However, according to the IPCC, a major gap in developing solutions is the lack of representation from vulnerable communities in the Global South (26). If the goals of the IPCC and United Nations Sustainable Development Goals are to be achieved, consistent and equitable engagement with communities experiencing the greatest vulnerability is critical (41–43).

## Inverting social innovation

Equitable engagement requires consistent community consultation to amplify the voices of disadvantaged citizens. To enable equitable engagement, communication between community members and intersectoral stakeholders is necessary for a comprehensive climate change response—a complicated process requiring extensive coordination among groups (44–46). Each stakeholder group also requires access to accurate data and evidence, which are essential for making informed and timely decisions for complex intersectoral problems such as climate change. Inaccurate and invalid data from unreliable sources can result in disjoint and ineffective decision-making (44)—an issue that became apparent in the inadequate response to climate disasters across the world in the summer of 2023 (45, 46). Although there is no silver bullet for climate change adaptation and mitigation, there is an imminent need for a methodology that would amplify citizen voices and enable effective decision-making across sectors. Such a solution potentially lies in ethically obtaining citizen-driven big data via their own ubiquitous digital devices (47).

Citizen-driven big data refers to data that are high in volume, velocity, variety, and veracity (48, 49). Ubiquitous digital devices, such as smartphones, generate big data through the use of embedded sensors (i.e., global positioning system and accelerometers) and digital platforms, which can play an important role in time-sensitive health crises including climate change. The use of digital technology can be considered a social innovation. The Organization for Economic Co-operation and Development defines social innovation as the design and implementation of new solutions to improve the wellbeing of individuals and communities, with a focus on economic development (50). Citizen-driven big data can transform responses to climate change risks across jurisdictions through the use of digital platforms. In particular, these platforms can amplify citizen voices for two key climate change adaptation and mitigation solutions: (1) Provide citizens with near real-time support and (2) Ethically relay citizen big data to decision-makers for rapid responses (51). Most importantly, digital platforms powered by citizen big data are not limited by jurisdictional boundaries, i.e., they can transform responses to climate change risks across jurisdictions—a potential paradigm-changing approach to social innovation. However, with historical injustices limiting current resource access for climate change adaptation and mitigation strategies in the Global South (28, 29), it is time to *invert* our approach to social innovation.

Thus, we define inverting social innovation as co-conceptualizing societal solutions with disadvantaged communities to improve economic development, with a focus on community wellbeing. For a community solution to be categorized as inverting social innovation, it should address a critical societal problem by satisfying three key criteria: (1) transform weaknesses into strengths, (2) prioritize citizen needs over corporate profits, and (3) provide a pathway for rapid scale-up across jurisdictions. Inverting social innovation does not minimize the importance of economic development but aims to amplify the voices of citizens to address existential public health and social crises such as climate change.

## Systems thinking

If there ever was an existential crisis that needed systems thinking to address its complexity, it is climate change (52, 53). Systems thinking is an approach to understanding and, ideally, solving complex problems that cut across disciplines and sectors. This holistic lens can potentially provide a pathway to develop and implement climate change adaptation and mitigation strategies which take into account the complexity of changing individual and contextual (i.e., social, ecological, economic, and political) risk factors. More importantly, these strategies may address determinants and risks within systems (e.g., food security—food supply within food systems) and across systems (e.g., food security and nutritional status across food and health systems) (54, 55). A systems thinking approach is essential for understanding the interdependent factors contributing to climate change and critical to identifying nodes of intervention within and across systems.

A significant challenge in monitoring, managing, and mitigating climate change impacts is the coordination of decision-making across systems (i.e., food, health, and social services) (54). The interdisciplinary expertise and trans-sectoral approaches that are required for coordinated decision-making can be transformed by ethically utilizing citizen-generated big data. Citizens’ lives do not exist in silos, so why do we think about solutions to societal problems in silos? Given that citizen-generated big data can cut across disciplines and sectors, systems thinking can be operationalized using digital citizen science (56). In the 21st century, human engagement with and through Internet-connected ubiquitous devices, such as smartphones, generates an enormous amount of big data (57). These big data can be used to understand and address complex, trans-sectoral problems. The use of big data can have significant positive implications for prediction, prevention (i.e., mitigation), and adaptation to climate change risks.

Digital platforms that are powered by citizen-driven big data offer a range of potential benefits, which include: (1) direct citizen-decision-maker communication to help mitigate and manage existing and emerging crises and (2) real-time support for citizens to improve their own decision-making during climate crisis (43). For example, citizens can obtain support in real time to help manage public health risks via an app that can predict evolving risks by taking into account both citizen as well as environmental big data. These big data can be aggregated and visualized on digital dashboards to facilitate jurisdiction-level rapid responses to evolving climate change impacts (e.g., direct public health crisis communication). The use of digital citizen science with systems thinking is a paradigm-changing approach that will invert social innovation, as it prioritizes community needs and, importantly, enables advancements in digital health for equity (43).

However, developing and implementing system-wide policies are extremely challenging without the timely collection, analysis, and visualization of data. It is evident that disparate information from multiple sectors can result in disjointed decision-making (26). To overcome this challenge in decision-making, it is imperative to adapt, implement, and evaluate existing digital platforms across jurisdictions, i.e., generate empirical evidence of their effectiveness. The operationalization (adaptation, piloting, implementation, and evaluation) of digital platforms for decision-making intersects multiple disciplines, including but not limited to computer science, data science, digital epidemiology, environmental sciences, global public health, implementation science, and program evaluation. This operationalization also transcends sectors, and can vary across jurisdictions, while maintaining standardized methods for rapid replication. However, to ensure the actual application of interdisciplinary and trans-sectoral approaches through systems thinking, it is critical to utilize previously tested and implemented frameworks that integrate citizen science with community-based participatory research via digital transformations (56).

One such framework is the Smart Framework, which has been consistently used to implement a range of innovative interventions, such as climate change adaptation (46, 58), co-creation of digital solutions for equity and justice (58), ethical surveillance promoting data sovereignty (59), and rapid jurisdictional decision-making (60). The application of the Smart Framework is driven by the integration of citizen science and community-based participatory research in addressing societal crises such as climate change. To enable this integration, the framework proposes repurposing citizen-owned internet-connected ubiquitous tools to sense, share, and link big data that power digital health platforms. In essence, the framework proposes that digital health platforms powered by citizen-driven big data can facilitate equity by amplifying disadvantaged citizen voices to inform policies—a key gap identified by the IPCC in current climate change solutions (26, 41–43). However, from a Global South perspective, as proposed by the Bridge Framework, systems thinking needs to go beyond sourcing big data from citizens to identifying processes for decolonizing citizen science to reverse historical injustices of colonization (61). Decolonizing citizen science is critical to ethically partnering with disadvantaged communities, where big data should aim to capture traditional local knowledge that is crucial for developing local solutions for global problems such as climate change. Hence, we propose the integration of concepts from both Smart and Bridge Frameworks to operationalize systems thinking via digital health platforms to address climate change impacts.

## Discussion

The foundation of citizen big data-driven digital health platforms for decision-making is co-creation (62). This involves the intersection of digital citizen science (i.e., direct engagement with all participants) and community-based participatory research action (i.e., direct engagement with jurisdictional decision-makers and leaders). The use of citizen-owned devices provides an avenue for big data collection, but perhaps more importantly, an opportunity to amplify citizen voices and empower those who experience marginalization to inform jurisdictional policies—a key component of co-creation (63). In addition to direct engagement with citizens, partnering with jurisdiction-specific Citizen Scientist Advisory Councils can facilitate concrete governance structures (47).

An efficient approach in developing digital health platforms for decision-making is to scale-up (43), replicate, and repurpose existing digital health infrastructure (64–67). Repurposing existing digital infrastructure aids rapid responses irrespective of the location of implementation—a capability that is critical for climate change adaptation and mitigation. Following this replicability-focused approach, emerging cutting-edge evidence indicates that the first step in developing and implementing digital health platforms for decision-making is to adapt existing cloud-based and *decentralized* digital infrastructure (51). An important aspect of this infrastructure is progressive web applications that can be built with web technologies, installed, and run on all digital devices or web browsers without the need to launch on Google and Apple stores (31). In essence, this decentralized approach can minimize dependency on big technology companies in responding to climate change (32, 51).

However, digital health platforms for climate change adaptation and mitigation must also enable real-time communication with citizens, particularly given the urgency of climate change events. This is a key component of enabling self-determination and sovereignty in the Global South (33). Such digital platforms can provide real-time support to households to manage key health risks within their jurisdiction by using advanced algorithms that will adapt to each household’s needs. All data from individual households can be encrypted, aggregated, and anonymized before being relayed to a digital decision-making dashboard to be housed with the key decision-makers in each jurisdiction. Decision-makers can use digital health dashboards to sustain real-time support for citizens and use big data for rapid responses (33, 51). For instance, decision-makers can send jurisdiction-specific disaster management alerts to mitigate and manage health risks, irrespective of the disaster (i.e., earthquakes, droughts, flooding, and forest fires) (51). The most advanced versions of these digital health dashboards can also support anonymous bi-directional engagement with citizens (51). In essence, these digital platforms can provide value perspective (i.e., real-time support) to citizens while amplifying citizen voices and contributing big data for a larger cause—jurisdictional responses for climate change adaptation and mitigation.

This value perspective is critical for the success of the infrastructure scale-up because it motivates citizens to participate in digital health platforms. Most importantly, the same digital health infrastructure could be modified to address jurisdiction-specific climate change issues ranging from infectious diseases (47) and non-communicable diseases (56) to systemic issues such as food security (68). This approach can transform the integration of health systems with other critical systems (i.e., environment, food, social services, and disaster management) to enable rapid responses for climate change adaptation and mitigation.

To replicate and scale-up digital health infrastructure for climate change adaptation and mitigation, equitable partnerships between decision-makers, researchers, and key community stakeholders in the Global South and North are paramount. To enable the meaningful inclusion of all research partners in the core decision-making processes of digital health infrastructure scale-ups, it is crucial to make a concerted effort to avoid top-down approaches that sometimes result when researchers in the Global North and South collaborate, i.e., a core systemic barrier to equitable partnerships (69, 70). Integrating digital citizen science with community-based participatory research (56, 61) can enable an equity-focused perspective to ensure meaningful partnership, participation, and contribution from all countries involved. The operationalization of this approach should start from the co-conceptualization stage itself, where representatives from each country are involved in establishing key risks, core objectives, data collection, and knowledge dissemination strategies. Since countries in the Global South continue to experience the most severe impacts of climate change, they should take the lead in informing digital health infrastructure adaptation and implementation.

The sustainability of digital health infrastructure will depend on training and development opportunities in each jurisdiction to essentially create a path for long-term capacity building. In implementing cutting-edge digital health infrastructure in the Global South, equitable technology transfer should be matched with the development of local technological skills. Ultimately, to implement digital health platforms, meaningful citizen involvement is key, particularly among vulnerable or marginalized communities experiencing the brunt of adverse climate change impacts. Thus, jurisdiction-specific Citizen Scientist Advisory Councils (68) can serve as co-creation and implementation partners, expanding the applicability of research findings and technology across society. Citizens interested in obtaining specific skills, such as interpretation of data, can be provided with training opportunities as part of efforts to build capacity. Given the disproportionate impacts of climate change on certain subgroups within communities (i.e., women and low-income households), successful implementation of digital health platforms also relies on their contribution to Citizen Scientist Advisory Councils.

Equitable Global South–North partnerships are the first step in ethically obtaining big data directly from disadvantaged citizens in the Global South (71, 72). These Equitable Global South–North partnerships can also lay the foundations for the data sovereignty of disadvantaged citizens in the Global South. As any digital ecosystem comes with risks of data exploitation, equitable partnerships can ensure that citizen data are secured within their jurisdictions by aligning with jurisdictional data regulations (51). However, to reverse historical injustices that the Global South continues to perpetuate (28, 29), we need to go above and beyond jurisdictional data legislation. One small step would be to use secure cloud computing to facilitate the data sovereignty of citizens, where citizens own their data (62).

Ultimately, if we are to transform health systems for climate change adaptation and mitigation in the global south, the three criteria for inverting social innovation provide a preliminary framework: (1) Transforming weakness into strengths: The 2022 IPCC report indicated a dearth of representation from disadvantaged communities in the development of climate change solutions. This gap can be addressed by decolonizing our approaches where diverse citizen voices are amplified via big data—an approach that has immense potential due to increasing ownership of not just ubiquitous devices such as smartphones, but also affordable access to data plans in the Global South (73–76). However, we cannot place all Global South countries in one box, as there are wide variations of access to mobile devices and data both within and across countries (77–79). Nevertheless, ubiquitous tools have transformative potential for health systems as well as people in the global south – if they are re-purposed ethically to utilize citizen-driven big data for climate change adaptation and mitigation; (2) Prioritizing citizen needs over corporate profits: Digital health platforms not only provide citizens with real-time support, but also relay aggregated and anonymized big data to jurisdiction-specific digital dashboards to enable decision-making, i.e., the needs of citizens and decision-makers of specific jurisdictions are prioritized; and (3) Providing a pathway for rapid scale-up across jurisdictions: The decentralized structure of the digital platforms for decision-making allows rapid scale-ups to adapt and mitigate climate change impacts specific to different jurisdictions, i.e., local solutions for global problems.

## Data availability statement

The original contributions presented in the study are included in the article/supplementary material, further inquiries can be directed to the corresponding author.

## Author contributions

TRK: Writing – original draft, Writing – review & editing, Conceptualization, Funding acquisition, Methodology. JB: Writing – original draft, Writing – review & editing, Conceptualization, Methodology.
